# Derivation and validation of a quantitative risk prediction model for weaning and extubation in neurocritical patients

**DOI:** 10.3389/fneur.2024.1337225

**Published:** 2024-02-27

**Authors:** Weiling Cheng, Ning Zhang, Dongcheng Liang, Haoling Zhang, Lei Wang, Leqing Lin

**Affiliations:** ^1^Department of Intensive Care Medicine, Hangzhou Normal University Affiliated Hospital, Hangzhou, China; ^2^Department of Biomedical Science, Advanced Medical and Dental Institute, Universiti Sains Malaysia, Penang, Malaysia

**Keywords:** traumatic brain injury, neurocritical care, weaning assessment, risk factors, risk prediction model

## Abstract

**Background:**

Patients with severe neurological conditions are at high risk during withdrawal and extubation, so it is important to establish a model that can quantitatively predict the risk of this procedure.

**Methods:**

By analyzing the data of patients with traumatic brain injury and tracheal intubation in the ICU of the affiliated hospital of Hangzhou Normal University, a total of 200 patients were included, of which 140 were in the modeling group and 60 were in the validation group. Through binary logistic regression analysis, 8 independent risk factors closely related to the success of extubation were screened out, including age ≥ 65 years old, APACHE II score ≥ 15 points, combined chronic pulmonary disease, GCS score < 8 points, oxygenation index <300, cough reflex, sputum suction frequency, and swallowing function.

**Results:**

Based on these factors, a risk prediction scoring model for extubation was constructed with a critical value of 18 points. The AUC of the model was 0.832, the overall prediction accuracy was 81.5%, the specificity was 81.6%, and the sensitivity was 84.1%. The data of the validation group showed that the AUC of the model was 0.763, the overall prediction accuracy was 79.8%, the specificity was 84.8%, and the sensitivity was 64.0%.

**Conclusion:**

These results suggest that the extubation risk prediction model constructed through quantitative scoring has good predictive accuracy and can provide a scientific basis for clinical practice, helping to assess and predict extubation risk, thereby improving the success rate of extubation and improving patient prognosis.

## 1 Introduction

With the continuous advancement of medical technology, mechanical ventilation, as an important treatment method in the field of critical care medicine, has been widely used in the treatment of neurocritical patients, significantly improving the survival rate of patients (1, 2). However, mechanical ventilation also brings many complications, such as pulmonary infection, tracheal injury, pulmonary hyperinflation, increased circulatory load, changes in hemodynamics, urinary tract infection, etc. Therefore, how to reasonably remove the endotracheal tube (extubation) is one of the urgent problems to be solved in the field of critical care medicine (3).

Due to the specificity of their conditions, the process of extubation for neurocritical patients is more complicated than for other patients, involving more risk factors (insufficient respiratory muscle strength, swallowing reflex disorders, pulmonary infection, atelectasis, intracranial hypertension, etc.) (4–7). Therefore, how to scientifically and reasonably assess the extubation risk of neurocritical patients and formulate appropriate extubation strategies has become an important content of clinical work (8).

Patients with severe neurological impairments frequently necessitate the provision of mechanical ventilation to sustain respiratory function, and the process of extubation necessitates a scaling back or cessation of this support, presenting a substantial challenge to the patient’s physiological resilience. These individuals frequently lack the autonomy to breathe independently, hence their reliance on ventilatory assistance; extubation, thus, demands the reinstatement of their self-sustaining respiratory capabilities, which exert significant pressure on the respiratory centers and associated musculature. In the context of severe neurological patients, determining the optimal moment for extubation can be a daunting task, as it involves precise ascertainment of the patient’s resumption of autonomous breathing and the assurance of a safe extubation process. Inappropriate timing of extubation can precipitate a decline in the patient’s health status, potentially culminating in life-threatening complications.

The contemporary paradigms within neurological intensive care lack robust empirical substantiation for the algorithmic management of airway interventions, such as intubation, extubation, and pneumatectomy. The existing randomized controlled trials (RCTs) and cohort investigations do not sufficiently mirror the patient demographic encompassing severe neurological pathologies (9–11). Consequently, the determinants heralding the success of extubation remain elusive, and the patient subset that might advantageously undergo direct tracheotomy (i.e., tracheotomy performed without prior extubation) is indeterminate. Scholarships that have endeavored to construct predictive indices for the likelihood of successful extubation have been hampered by methodological constraints, including the enrollment biases of single-center studies and the absence of a validation cohort (12). Hence, there exists a paucity of evidence-informed clinical guidelines to direct the clinical practice of tracheal extubation and tracheotomy in patients afflicted with severe neurological ailments.

Cinotti et al. have disseminated the outcomes of the Extubation Strategy and Prognosis (ENIO) study conducted among patients within neurointensive care units (13). The study found that 19.4% of patients who underwent extubation experienced failure of the extubation process within a 5-day window (serving as the primary endpoint). Extubation failure was observed to be less prevalent among youthful patients, those suffering from Traumatic Brain Injury (TBI), and individuals exhibiting optimal levels of alertness. Respiratory insufficiency was identified as a more common rationale for reintubation than instances of airway or nervous system failure (14). Additionally, a further 21.1% of patients ultimately underwent direct tracheotomy, subsequent to a median interval of 9 days (with a range of 5 to 15 days, as indicated by the interquartile range) (15).

In recent annums, a cadre of studies has been endeavored to prognosticate the likelihood of extubation failure among neurological patients. In the absence of a proprietary extubation metric tailored for patients with Traumatic Brain Injury (TBI), the VISAGE score has been predominantly adopted within the broader population of neurology intensive care units, factoring in laryngeal reflexes, cough efficacy, sputum expectoration, and the neurological status as gaged by the visual subscale of the revised Coma Recovery Scale (16). Nonetheless, this scoring system necessitates external validation. Indeed, the extant corpus of research includes investigations into the prevalent scenarios and determinants of extubation failure. Current methodologies, however, lack a systematic appraisal of the precision and relative accuracy of extubation risk prediction models for craniocerebral injured patients, precluding the empirical endorsement of extant risk prediction models. This limitation hampers the clinical application of precise and pertinent risk assessment methodologies. It is imperative for subsequent scholarly inquiries to delve into the pathophysiology of extubation failure in cerebral-injured patients, thereby ameliorating the risk profile for those susceptible to extubation failure and forestalling subsequent reintubation. Establishment of a risk prediction model that can objectively ascertain risk and augment the success rate of extubation is a critical imperative. This research is dedicated to constructing a predictive model and scoring system for extubation, that will quantitatively assess the suitability of tracheal intubation patients with craniocerebral injury in the intensive care unit for extubation, as well as the influence of pertinent factors. The study aims to furnish practical guidance for the prevention of post-extubation complications, thereby mitigating the incidence of extubation-related failures.

Although there are currently some studies exploring the risk factors for extubation in critically ill patients, most of these studies are single-center and small-sample studies, and the generalizability and accuracy of their results remain to be further verified. Therefore, this study intends to explore the actual situation and related risk factors of extubation in ICU neurocritical patients with mechanical ventilation through evidence-based methods, screen out independent risk factors, and construct a quantitative extubation risk prediction model based on these factors to provide a more scientific and reasonable basis for clinical extubation decision-making. At the same time, this study will also strictly validate the constructed risk prediction model to ensure its accuracy and reliability in clinical practice (17). The main objective of this study was to develop and validate a quantitative risk prediction model for predicting risk during withdrawal and extubation in patients with severe neurological conditions.

## 2 Materials and methods

### 2.1 Study subjects

A total of 200 critically ill neurology patients admitted to the Intensive Care Unit of our hospital from January 2020 to December 2022, and who underwent invasive mechanical ventilation for more than 24 h, were selected for this study. This study was approved by the Ethics Committee of Hangzhou Normal University.

#### 2.1.1 Inclusion criteria

Neurocritical patients: TBI, SAH, intracranial hemorrhage (ICH), ischemic stroke, and central nervous system infections (brain abscess, ventriculitis, meningitis, encephalitis, or brain tumors), etc., who completed the acute phase treatment in the ICU, and meet the following criteria: (1) Aged ≥18 years, admitted to the ICU with a GCS <12 before endotracheal intubation, requiring invasive mechanical ventilation for ≥24 h and have attempted extubation (i.e., underwent an extubation trial) and/or tracheostomy.

#### 2.1.2 Exclusion criteria

(1) Age < 18 years old; (2) Pregnant or lactating patients at the time of onset; (3) Spinal cord injury above T4, cardiac arrest resuscitation, Guillain-Barre syndrome, motor neuron disease, myopathy and severe myasthenia gravis, death before extubation, end-of-life extubation, withdrawal of life-sustaining treatment (WLST) within 24 h of ICU admission, respiratory system complications (long-term home oxygen therapy, chronic obstructive pulmonary disease Gold grade III or IV), and severe chest trauma (Abbreviated Injury Score, AIS ≥ 3); (4) Patients with tracheostomy before ICU admission are also excluded; (5) Patients who have never been weaned from IMV are not included; (6) Patients with incomplete clinical data.

### 2.2 Sample size

*N* = *Z*^2^ × [P × (1-P)]/*E*^2^, the confidence interval *Z* is set to 1.96, the probability value *P* is set to 90%, and the allowable error is set to less than 10%. It is concluded that the sample size required for this study is at least 60–80 cases. Considering that the success rate of extubation in ICU patients with traumatic brain injury is approximately 62% (14), the minimum sample size required for modeling is 96–129 cases. In this study, there were 140 patients in the modeling group, meeting the sample size requirement for modeling. At the same time, to ensure that there is a sufficient sample size to obtain the risk factors for extubation, the ratio of the sample size of the modeling group to the validation group was set at 7:3 (18). This not only ensures a sufficiently large modeling sample size but also avoids the problem of excessive sampling error due to the small number of data in the validation group. Therefore, the total sample size for this study is 200 cases, which is sufficient to ensure the reliability and accuracy of the research results. This investigation employed a stratified randomization technique to allocate subjects to diverse treatment cohorts. Stratified randomization is a strategy devised to enhance the integrity of randomization by dividing participants based on recognized pivotal variables (such as age, gender, body mass index) and subsequently randomizing within each stratum to guarantee an equitable distribution of these variables across the treatment groups within each stratum. To ascertain that the randomization remains balanced throughout the process, the distribution of treatment groups within each stratum and collectively is periodically scrutinized to detect any substantial imbalances.

### 2.3 Data collection

The data collection work includes three aspects: demographic and baseline data, extubation-related data, and prognosis-related data. Demographic and baseline data mainly include age, gender, baseline Glasgow Coma Scale (GCS), BMI, location of brain injury, comorbidities (such as diabetes, hypertension, chronic lung disease, etc.), and the Acute Physiology and Chronic Health Evaluation (APACHE II) within 24 h of ICU admission.

Extubation is a critical procedure within the domain of intensive care. Its successful execution necessitates a meticulous evaluation of various physiological indices, the acuity of the illness, respiratory competence, coughing efficiency, and the presence of airway edema. Throughout the extubation process, a battery of standardized tests and clinical assessments are employed to gage the viability of extubation and to anticipate potential post-extubation complications. These include, but are not limited to:

*Spontaneous Breathing Test (SBT)*: This test evaluates a patient’s capability to breathe independently of mechanical ventilation. It serves to assess the strength and synchronization of respiratory muscles. The success of an SBT is commonly correlated with parameters such as the peak expiratory flow rate of cough (C-PEFR), and diaphragmatic function.

*Balloon Leak Test (BLT)*: This test is utilized to determine the extent of airway edema and to verify airway patency by observing whether the patient is able to breathe normally following deflation of the tracheal intubation balloon.

*Diaphragmatic Ultrasound*: As the diaphragm is the primary respiratory muscle, its movement and contraction rate can be monitored via ultrasound, thereby providing an indirect assessment of the patient’s respiratory function.

Clinical Scoring Systems including the Visual Inspiratory Sniffing Angle (VISAGE) Score and the Respiratory Insufficiency Intubation Score (RIS-1): These scoring systems integrate multiple clinical variables to offer a holistic evaluation of a patient’s risk profile during extubation. SBT and diaphragmatic function tests primarily reflect the patient’s respiratory muscle function, while the BLT and airway patency assessments are centered on identifying airway-related risks. The VISAGE and RIS-1 scores encapsulate the patient’s general health status and the risks associated with extubation.

Extubation-related data mainly includes records of successful spontaneous breathing trials (SBT), the date of the first extubation attempt or tracheotomy, and general management data collected on the day of extubation, such as the use of corticosteroids (to prevent wheezing after extubation) or the interruption of enteral nutrition, swallowing function, duration of tracheal intubation, cough reflex, sputum volume, and lung infection status. Standardized clinical examination on the day of extubation: vital signs (body temperature, heart rate, systolic blood pressure), breathing (including the type and time of SBT), physical examination (cough assessment, GCS eye language movement items, vomiting reflex). The definitions of these features were standardized based on previously described data. For example, a 4-level scale was used to assess cough intensity: severe, moderate, weak, and none. Record the time and reason for re-intubation. The swallowing function assessment uses the Standardized Swallowing Assessment (SSA) to screen patients for swallowing disorders before extubation. The scale consists of three parts, ranging from easy to difficult: preliminary clinical examination, 5 mL water test, and 60 mL water test. If any item is abnormal during the test, the test is immediately terminated, and the patient is judged to have a positive swallowing disorder. The score of this scale is negatively correlated with the patient’s swallowing function, with a minimum score of 18 and a maximum score of 46. Prognosis-related data mainly includes ICU length of stay, total length of hospital stay, and clinical outcome (death/transfer, etc.).

The extubation test usually refers to a series of examinations and assessments to assess a patient’s suitability for removal of the tracheal tube, which may include airway patency, the patient’s respiratory function, coughing ability, and swallowing ability. Swallowing grading usually refers to a method of assessing a patient’s ability to swallow, and it can help determine if the patient has dysphagia, which is related to whether the patient can eat and water safely after extubation. Swallowing grading may include observing the patient’s mouth and throat movements while swallowing, as well as assessing whether there is a risk of aspiration of food or fluid after swallowing. The cough reflex refers to a patient’s ability to clear foreign objects or secretions from the airway by coughing. This is an important defense mechanism for maintaining airway patency and is often evaluated before and after extubation. These assessment methods, including SBT, extubation tests, swallowing grading, and cough reflex assessment, are also applicable in neurosurgical patients. Since neurosurgical patients may be affected by surgical trauma, disturbance of consciousness, dysphagia, and other factors, these assessments are even more important because they can help physicians determine the safety of patients after extubation and possible risk of complications. Anesthetics may affect the evaluation of extubation tests. Anesthesia can affect a patient’s respiratory center, muscle strength, and coordination, so extubation tests performed during or immediately after anesthesia withdrawal may not accurately reflect a patient’s ability to breathe on his or her own in a fully awake state. Therefore, when evaluating extubation tests, we need to take into account the patient’s current state of anesthesia and evaluate after the patient is awake and the effects of anesthesia have subsided to obtain more accurate results.

### 2.4 Establishment and grouping of model datasets

#### 2.4.1 Modeling method

The data of 200 cases in the modeling group database were used to construct the model. Taking whether the extubation was successful as the dependent variable, univariate analysis was performed on general and clinical data that may affect the extubation of ICU patients with tracheal intubation. Before conducting the logistic regression analysis, tolerance and variance inflation factor (VIF) tests were used to check whether there was multicollinearity among the variables. Then, each variable was included in the binary logistic regression analysis according to the entry standard of 0.05 and the removal standard of 0.10 to further determine the independent risk factors of PED. The partial regression coefficient values, 95% confidence interval, and OR values of each independent risk factor were obtained through binary logistic regression analysis, and the calculation formula of the risk prediction model was obtained. Based on the partial regression coefficient values of each indicator in the logistic regression analysis model, a score can be assigned to each indicator, thereby establishing a risk scoring model for extubation in ICU patients with tracheal intubation, which is convenient for clinical use. The specific steps are as follows: Divide the partial regression coefficient of each variable by the smallest regression coefficient value in the logistic regression analysis model, then multiply by a constant 2, and finally round to obtain the score of that variable. In this way, the PED risk scoring model can be established. According to the scoring standard of the predictive model, the scores can be substituted into the regression equation, and the ROC curve of risk scoring can be plotted. The sensitivity and specificity at different scores can be obtained from the curve, and the Youden index (sensitivity + specificity − 1) can also be calculated from the curve. By maximizing the Youden index, the critical point can be determined for risk assessment.

#### 2.4.2 Validation method

To verify the accuracy and clinical applicability of the extubation risk prediction model, we evaluated the model using 140 validation group data from the database. Firstly, the variables of the validation group were input into the established extubation risk prediction model to calculate the risk of extubation failure. Then, we evaluated the model using three indicators: discrimination, consistency, and clinical utility. Discrimination reflects the model’s ability to differentiate whether patients will undergo re-intubation, typically evaluated using the C-statistic. The C-statistic is the area under the ROC curve (AUC), with values closer to 1 indicating better predictive accuracy. Generally, a C-statistic greater than 0.7 indicates good predictive ability, while a C-statistic greater than 0.8 indicates excellent predictive ability. Consistency reflects the agreement between the predicted probabilities and actual probabilities, usually assessed using the Hosmer-Lemeshow (H-L) goodness-of-fit test. A p-value greater than 0.05 indicates good consistency. Clinical utility refers to the guidance value of the model in categorizing patients into low-risk and high-risk groups, aiming to help clinicians decide whether preventive interventions are necessary. Setting an appropriate threshold can balance the need for necessary interventions and avoid unnecessary ones. In this study, we determined the sensitivity and specificity at different scores according to the scoring standard of the predictive model and ROC curve, and identified the critical point by calculating Youden’s Index (YI).

### 2.5 Statistical analysis

Data were analyzed using SPSS 26.0. For data that followed a normal distribution, mean ± standard deviation (mean ± SD) was used to express the results; for non-normally distributed data, median and interquartile range (IQR) were used. Analysis of variance (ANOVA) was employed to test the differences among groups, Mann–Whitney U test was used for between-group comparisons with unequal variances, and *t*-test was used for comparisons between groups. Logistic regression analysis and other statistical methods were also applied. A *p*-value less than 0.05 was considered statistically significant.

## 3 Results

### 3.1 Clinical data

In this study, we collected data on 200 ICU patients who met the inclusion criteria. Among them, 61 cases (43.6%) were spontaneous intracerebral hemorrhages, 73 cases (52.1%) were traumatic intracerebral hemorrhages, and 6 cases (4.3%) were ischemic strokes. The median age was 54 years old (ranging from 19 to 91 years old), and the baseline GCS score was 12 points (ranging from 3 to 15 points). Using a random number table method, 60 patients were selected for validation of the extubation risk prediction model (validation group). The construction of the risk prediction model (modeling group) was validated with the remaining 140 patients. Table 1 shows the general information and clinical characteristics of the patients in the derivation group, of which 115 cases (82.1%) were male, 25 cases (17.9%) were female, with an average age of 63 years old (ranging from 19 to 91 years old). 45.7% of patients were aged ≥65 years old; 15.7% of patients had a BMI <18.5, and 20% had a BMI ≥24. The APACHE II score within 24 h of ICU admission was 13.12 ± 3.75, with 33.6% of patients scoring ≥15. 77.1% of patients primarily underwent surgeries such as hematoma removal, decompressive craniectomy, and stereotactic puncture drainage. The length of ICU stay was 10.48 ± 5.26 days, and the total hospital stay was 28.54 ± 6.15 days. Finally, Table 1 also shows the general situation and clinical characteristics of patients in the verification group.

**Table 1:** General information and clinical characteristics of patients (derivation, *n* = 140 and Validation group, *n* = 60).

Variable name	Variable classification	Frequency (cases)	Percentage (%)	Frequency (cases)	Percentage (%)
		Derivation group		Validation group	
Reason for admission	Spontaneous intracerebral hemorrhage	61	43.6	25	41.7
	Traumatic intracerebral hemorrhage	73	52.1	34	56.7
	Ischemic stroke	6	4.3	1	1.7
Age (years)	<65	87	62.1	23	38.8
	≥65	53	37.9	37	61.2
Gender	Male	115	82.1	48	80
	Female	25	17.9	12	20
BMI	<18.5	22	15.7	7	11.7
	18.5–23.9	90	64.3	37	61.7
	≥24	28	20	16	26.7
APACHE II score	<15	79	56.4	34	56.7
Medical history	Diabetes	33	23.6	8	13.3
	Hypertension	92	65.7	72	53.3
	Coronary heart disease	14	10.0	7	11.7
	Cerebrovascular disease	13	9.3	5	8.3
	Chronic pulmonary disease	15	10.7	8	13.3
	Other	21	15	8	13.3
Surgical treatment	Yes	111	79.3	48	80
	No	29	20.7	12	20
ICU Length of Stay (days)	≤5	24	17.1	10	16.7
	6 ~ 10	53	37.9	18	30.0
	11 ~ 15	34	24.3	11	18.3
	≥15	29	20.7	21	35.0
Total hospital length of stay (days)	≤10	14	10	7	11.7
	11 ~ 20	26	18.6	10	16.7
	21 ~ 30	44	31.4	10	16.7
	≥30	56	40	33	55.0
Clinical outcome	Improved	77	55.7	36	60.0
	Not cured	35	25	5	8.3
	Deceased	28	20	19	31.7

### 3.2 Univariate regression analysis

Before conducting regression analysis, continuous variables in this study were transformed into categorical variables according to literature review and clinical experience to make the predictive model more convenient for clinical use. Factors that may be significantly correlated with successful extubation include general characteristics of the patients such as age, APACHE II score, BMI, etc.; GCS score, oxygenation index on the day of extubation; airway characteristics including cough reflex, frequency of tracheal suctioning, and swallowing function (Table 2).

**Table 2:** Variables assignment table for analysis of extubation risk factors in patients with craniocerebral injury.

Variable name	Assignment	Variable type
Age	1 = <65 years old; 2 = ≥65 years old	Binary variable
Gender	1 = Male; 2 = Female	Binary variable
BMI	1 = <18.5 kg/m^2^; 2 = 18.5 ~ 23.9 kg/m^2^; 3 = ≥24.0 kg/m^2^	Ordinal variable
APACHE II score	1 = <15 points; 2 = ≥15 points	Binary variable
Diabetes	Yes/No (1 = Yes; 0 = No)	Binary variable
Hypertension	Yes/No (1 = Yes; 0 = No)	Binary variable
Coronary heart disease	Yes/No (1 = Yes; 0 = No)	Binary variable
Cerebrovascular Disease	Yes/No (1 = Yes; 0 = No)	Binary variable
COPD	Yes/No (1 = Yes; 0 = No)	Binary variable
Surgical treatment	Yes/No (1 = Yes; 0 = No)	Binary variable
Smoking history	Yes/No (1 = Yes; 0 = No)	Binary variable
Use of steroids before extubation	Yes/No (1 = Yes; 0 = No)	Binary variable
Enteral nutrition interruption	Yes/No (1 = Yes; 0 = No)	Binary variable
Sputum volume (suctioning frequency)	3= > 3time/h；2 = 2 ~ 3time/h；1 = ≤1time/h	Ordinal variable
Cough strength	3 = Strong；2 = Moderate；1 = Weak	Ordinal variable
Swallowing function (SAA Score)	4 = 18 ~ 24；3 = 25 ~ 31；2 = 32 ~ 38；1 = 39 ~ 46	ordinal variable
SBT test	1 = Pass; 0 = Fail	Binary variable
Leak test	1 = Pass; 0 = Fail	Binary variable
Oxygenation index	0 ≥ 300; 1<300	Binary variable
Heart rate	1 = <60 beats/min; 2 = 60–100 beats/min; 3 = >100 beats/min	Ordinal variable
GCS score	1 = <8 points; 2 = 8 ~ 15 points	Binary variable
Respiratory rate	1 = <18 breaths/min; 2 = ≥18 breaths/min	Binary variable
Body temperature	1 = <36.5°C; 2 = 36.5–36.9°C; 3 = 37–37.9°C; 4 = ≥38°C	Ordinal variable

For whether the extubation was successful or not as the dependent variable, single-factor analysis was conducted on the general and clinical data that may affect critically ill neurologic patients and mechanically ventilated patients undergoing extubation. The results are shown in Tables 3, 4. Among the patients, 113 cases (80.7%) attempted extubation at least once, and 5 cases (3.6%) required reintubation within 48 h after extubation. In patients with extubation failure, there were fewer cases of spontaneous intracerebral hemorrhage [22 cases (52.4%) vs. 39 cases (39.8%), *p* = 0.02], older age [61 years old (19–91 years old) vs. 54 years old (20–89 years old), *p* = 0.001], and lower GCS scores on the day of extubation [7 points (5–8 points) vs. 7 points (5–9 points), *p* = 0.003]. Details are shown in Table 3.

**Table 3:** Single-factor analysis of general information for the two groups [case (%)/M (Q)].

Variable name	Stratification	Successful extubation group	Failed extubation group	x^2^	p-value
		n = 98	n = 42		
Reason for admission	Spontaneous intracerebral hemorrhage	39 (39.8%)	22 (52.4%)		0.2
	Traumatic intracerebral hemorrhage	54 (55.10%)	19 (45.2%)		0.06
	Ischemic stroke	5 (5.1%)	1 (2.4%)		0.4
General characteristics					
Age	>65 years old	27 (27.6%)	26 (61.9%)	11.685	0.001**
	≤65 years old	71 (72.4%)	16 (38.1%)		
Gender	Male	81 (82.7%)	33 (78.6%)	0.118	0.732
	Female	17 (17.3%)	9 (21.4%)		
Smoking history	Yes	25 (25.5%)	7 (16.7%)	0.574	0.751
	No	73 (74.5%)	36 (83.3%)		
BMI	<18.5	15 (15.3%)	7 (16.7%)	4.447	0.108
	18.5 ~ 23.9	66 (67.3%)	23 (54.8%)		
	≥24.0	17 (17.4%)	12 (28.5%)		
APACHEII score	<15 points	42 (42.9%)	37 (88.1%)	6.734	0.009**
	≥15 points	56 (57.1%)	5 (11.90%)		
Surgical treatment	Yes	81 (82.7%)	30 (71.4%)	1.341	0.247
	No	17 (17.3%)	12 (28.6%)		
Past medical history					
Diabetes	Yes	12 (12.2%)	5 (11.9%)	0.46	0.498
	No	86 (87.8%)	37 (88.1%)		
Hypertension	Yes	56 (57.1%)	22 (52.4%)	0.194	0.66
	No	42 (42.9%)	21 (47.6%)		
Coronary heart disease	Yes	9 (9.2%)	5 (11.9%)	3.758	0.053
	No	89 (90.8%)	37 (88.1%)		
Cerebrovascular disease	Yes	7 (7.1%)	4 (9.5%)	0.378	0.539
	No	91 (92.9%)	38 (90.5%)		
Chronic pulmonary disease	Yes	6 (6.1%)	9 (21.4%)	4.879	0.027*
	No	92 (93.9%)	33 (78.6%)		

**Table 4:** Univariate analysis of different characteristics between two groups [case (%)].

Variable name	Stratification	Successful extubation group	Failed extubation group	x^2^	p-value
		n = 98	n = 42		
Days of endotracheal intubation		6 [3–14]	7 [5–17]		0.539
SBT test	T-tube oxygenation	59 (60.2%)	27 (64.3%)	0.569	0.451
	CPAP mode	39 (39.8%)	15 (25.7%)		
Days from SBT to extubation		0 [0–2]	1 [0–2]		0.4
Assessment on the day of extubation					
SBT test	Passed	98 (100%)	35 (83.3%)	3.106	0.078
	Failed	0 (0%)	7 (16.7%)		
Leak test	Passed	96 (97.9%)	38 (90.5%)	3.237	0.072
	Failed	2 (2.1%)	4 (9.5%)		
Steroid use	Passed	41 (41.8%)	18 (42.9%)	0.025	0.873
	Failed	57 (58.2%)	24 (57.1%)		
Enteral nutrition interruption	Yes	72 (73.5%)	30 (71.4%)	1.783	0.209
	No	26 (26.5%)	12 (10.6%)		
Oxygenation index	≥300	89 (90.8%)	28 (66.7%)	8.105	0.004**
	<300	9 (9.2%)	14 (33.3%)		
Heart rate (beats/min)	<60	22 (22.4%)	9 (21.4%)	0.015	0.904
	60–100	59 (60.2%)	20 (47.6%)		
	>100	17 (17.4%)	13 (31.0%)		
Temperature (°C)	<36.5	12 (12.2%)	4 (9.6%)	0.508	0.476
	36.5–36.9	41 (41.8%)	15 (25.7%)		
	37–37.9	31 (31.6%)	15 (25.7%)		
	≥38.0	14 (14.3%)	8 (19.0%)		
Respiratory rate (breaths/min)	<18	69 (70.4%)	25 (59.5%)	0.088	0.767
	≥18	29 (29.6%)	17 (40.5%)		
GCS score (points)	<8 points	16 (16.3%)	33 (78.6%)	8.604	0.003**
	≥8 points	82 (83.7%)	9 (21.4%)		
Sputum volume (suction frequency)	>3 time/h	13 (13.3%)	24 (57.1%)	4.826	0.028*
	2-3 time/h	31 (31.6%)	10 (23.8%)		
	0-1 time/h	54 (55.1%)	8 (19.1%)		
Cough strength	Severe	58 (59.2%)	7 (16.7%)	4.104	0.043*
	Moderate	31 (31.6%)	20 (47.6%)		
	Weak	9 (9.2%)	15 (25.7%)		
Swallowing function (SSA score)	18 ~ 24	2 (2.0%)	18 (42.9%)	17.147	0.001**
	25 ~ 31	9 (9.2%)	12 (28.6%)		
	32 ~ 38	28 (28.6%)	8 (19.0%)		
	39 ~ 46	59 (60.2%)	4 (9.4%)		

The data of 140 patients was used to construct the model. The single-factor analysis results of the general data of the two groups showed that age, APACHEII score, and whether there was a combination of COPD had statistical significance (all *p* < 0.05), as shown in Table 3. Age ≥ 65 years old (2 = 11.685, *p* = 0.001), APACHEII score ≥ 15 points (2 = 6.734, *p* = 0.009), and the combination of chronic lung disease (2 = 4.879, *p* = 0.027) are potential risk factors for extubation failure. As age increases, the possibility of extubation failure tends to increase. As the APACHEII score increases, especially in patients with scores ≥15, the incidence rate of extubation failure increases. Patients with combined chronic lung disease have a higher proportion of eventual extubation failure clinically. The single-factor analysis results of the two groups with different characteristics showed that the GCS score on the day of extubation, sputum volume, cough strength, and swallowing function were statistically significant (all *p* < 0.05), as shown in Table 4.

### 3.3 Multivariate regression analysis

Tolerance and variance inflation factor (VIF) were used to test for multicollinearity among variables prior to logistic regression analysis to determine if there is multicollinearity among variables. Based on the standard proposed by Kleinbaum DG, the criterion for the presence of multicollinearity is a tolerance of <0.1 or a VIF of >10.0 (19). In this study, there was no multicollinearity among the variables. The dependent variable is whether the extubation is successful, with an entry standard of 0.05 and a removal standard of 0.10. The meaningful variables in the univariate analysis: age ≥ 65 years (*p* = 0.001), APACHE II score ≥ 15 points (*p* = 0.009), combined chronic pulmonary disease (*p* = 0.027), etc., were included in the equation by the stepwise forward method (Likelihood Ratio, LR: forward) of the maximum likelihood ratio estimate. Table 5 shows the results of the binary logistic regression analysis. The equation itself is meaningful (*χ*^2^ = 46.806, *p* < 0.001), NagelkerkeR2 = 0.401. Seven risk factors affecting whether the patient is successfully extubated entered the Logistic regression equation, namely age ≥ 65 years, high APACHE II score (≥ 15 points), chronic pulmonary disease, GCS score on the day of extubation, sputum volume (sputum suction frequency), cough reflex, and swallowing function. As age increases, the risk of extubation failure in ICU patients increases; simultaneously, as the severity of the patient’s disease increases, such as an increase in the APACHE II score, the incidence of extubation failure also increases, that is, the higher the severity of the disease, the higher the incidence of extubation failure; if chronic pulmonary disease is combined, the possibility of extubation failure increases; on the day of extubation, the results of the blood gas analysis of the SBT test, if the oxygenation index is less than 300, then re-intubation or tracheostomy is required after extubation. In addition, if the patient is in a deeper coma, with a GCS score of <8, and at the same time has a large amount of sputum, poor cough reflex, and swallowing dysfunction, it will greatly increase the failure rate of extubation.

**Table 5:** Multifactorial logistic regression analysis of risk factors for extubation in patients with mechanical ventilation.

Risk factor	Bias regression coefficient	Standard	p-value	OR value	95%CI
Elderly (≥65 years old)	1.374	0.370	0.000	3.95	1.912–8.159
High APACHEII score (≥15 points) (≥15 分)	1.518	0.409	0.000	4.563	2.046–10.176
Chronic lung disease	0.971	0.395	0.014	2.64	1.218–5.724
Characteristics on the day of extubation					
GCS score	1.038	0.507	0.041	2.824	1.045–7.633
Oxygenation index	2.020	0.381	0.000	7.538	3.575–15.892
Airway mucus volume	1.225	0.384	0.001	3.404	1.605–7.220
Cough strength	0.873	0.250	0.000	2.394	1.468–3.905
Swallowing function	1.170	0.420	0.005	3.223	1.414–7.345
Constant	−4.330	0.557	0.000	0.013	

### 3.4 Establishment of risk prediction model

In this cohort, 140 patients were available for constructing a scoring system to predict successful extubation. The simplified score retained 8 predictive factors (Supplementary material 1): age, APACHE II score, comorbid chronic pulmonary disease, oxygenation index, severe cough, swallowing function, suction frequency, and GCS score total. Based on the partial regression coefficients of the above variables, the ICU post-extubation dysphagia risk prediction model formula was constructed as follows: Risk of extubation failure = $\frac{e^{a}}{1+e^{a}}\times 100\%$ , where *e* is the exponential function. *a* = 1.374 × age (= 0 or 1) + 1.518 × APACHE II score (= 0 or 1) + 0.971 × comorbid chronic pulmonary disease (= 0 or 1) + 1.038 × GCS score (= 0 or 1) + 2.020 × oxygenation index (= 0 or 1) + 1.225 × airway sputum volume [hourly suction frequency (= 1 or 2 or 3)] + 0.873 × cough intensity (= 1 or 2 or 3) + 1.170 × swallowing (SSA score) (= 1 or 2 or 3) − 4.330.

Based on the logistic regression analysis model, we assigned corresponding scores to each indicator’s partial regression coefficient, making the ICU extubation risk scoring model for severe neurological patients easier to use in clinical practice. Specifically, for each variable, we divided its partial regression coefficient by the smallest regression coefficient value in the logistic regression analysis model (0.971), then multiplied it by a constant 2, and rounded to the nearest integer (20). The final scores for each variable are shown in Table 6. The scores for age < 65, APACHE II score < 15, no history of chronic pulmonary disease, GCS score ≥ 8, oxygenation index ≥300, suction frequency, cough intensity, and swallowing function are 3, 3, 2, 3, 3, 2, 3, and 4, respectively.

**Table 6:** Scoring criteria for the Logistic model of extubation risk in mechanically ventilated patients.

Risk factors		Score
Advanced age (≥65 years) (NO)		3
High APACHE II score (≥15 points) (NO)		3
Chronic pulmonary disease (NO)		2
Characteristics on the day of extubation		
GCS score ≥ 8 points (Yes)		3
Oxygenation index ≥300 (Yes)		3
Airway secretions (suction frequency)	≥3 times/h	0
	2 times/h	1
	1 time/h	2
Cough strength	Severe	3
	Moderate	2
	Weak	1
Swallowing function (SSA score)	18 ~ 24	1
	25 ~ 31	2
	32 ~ 38	3
	39 ~ 46	4

The scoring criteria of the prediction model were substituted into the regression equation, and the ROC curve was drawn. The sensitivity and specificity at different scores can be obtained from this curve, and Youden’s index (sensitivity + specificity − 1) can also be calculated from this curve. By maximizing Youden’s index, the critical point can be determined for risk assessment. Table 7 shows that the highest Youden’s index total score is 18 points. Therefore, the critical value of this study model is set at 18 points. A score of less than 18 suggests that the risk of extubation failure may be higher, and a tracheotomy may be clinically recommended. Patients with a score of 18 or more have a relatively higher success rate of extubation and may try to wean off the ventilator.

**Table 7:** Sensitivity and specificity of the predictive model at different cutoff values.

Total score	Sensitivity	Specificity	Youden index
3	1.000	0.000	0.000
4	1.000	0.000	0.000
5	1.000	0.000	0.000
6	1.000	0.092	0.092
7	0.969	0.138	0.107
8	0.938	0.345	0.283
9	0.922	0.420	0.342
10	0.911	0.534	0.445
11	0.906	0.583	0.565
12	0.898	0.604	0.502
13	0.875	0.629	0.657
14	0.864	0.641	0.505
15	0.862	0.705	0.507
16	0.853	0.712	0.565
17	0.849	0.724	0.573
18	0.841	0.816	0.657
19	0.703	0.816	0.519
20	0.652	0.855	0.507
21	0.516	0.861	0.377
22	0.492	0.919	0.411
23	0.313	0.931	0.244
24	0.250	0.951	0.201
25	0.247	0.954	0.201
26	0.215	0.977	0.377
27	0.186	0.983	0.411
28	0.142	0.994	0.244
29	0.094	1.000	0.201
30	0.031	1.000	0.094
31	0.000	1.000	0.031
32	0.000	1.000	0.000
33	0.000	1.000	0.000

**Figure 1:**
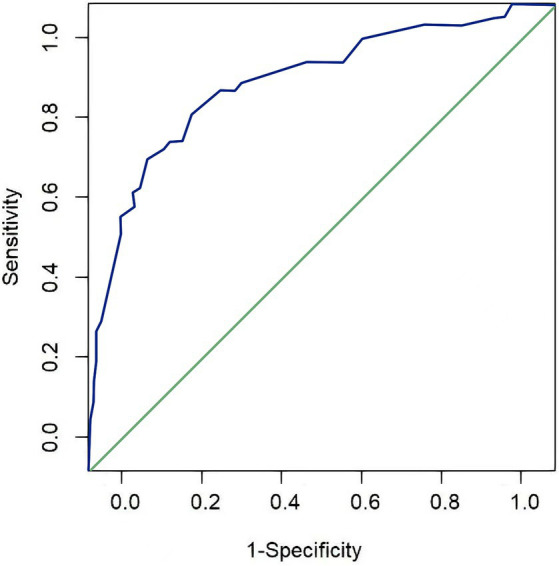
ROC curve of the risk prediction model in the modeling group.

To evaluate the established risk prediction model for extubation failure, the ROC curve method and the H-L goodness-of-fit test were used to test the discrimination and consistency of the model. The results of the H-L goodness-of-fit test showed that there was no statistical difference between the predicted incidence rate and the actual incidence rate of the model (*x*^2^ = 5.241, *p* = 0.732, *p* > 0.05), indicating that the prediction model has good consistency. Figure 1 is the ROC curve of the extubation risk prediction model. The area under the curve (AUC), or C-statistic, is 0.832 (95% CI, 0.770–0.894), which is close to 1, indicating that the model can well distinguish whether the patients can be successfully extubated. At a score of 18, the specificity of the ROC curve of this model is 81.6%, and the relative sensitivity is 84.1%. The overall prediction accuracy rate is 81.5%, and the negative predictive value is 90.8%, which is relatively high, while the positive predictive value is relatively low at 66.3%, which may be due to the small number of positive samples (only 42 cases). Therefore, to further verify the predictive performance of the model, it is necessary to increase the sample size.

### 3.5 Validation of risk prediction model

Table 8 shows the distribution of clinical variables in the two groups. There was no statistically significant difference in clinical variables between the two groups.

**Table 8:** Distribution of clinical variables in the modeling group and validation group.

variables	Stratification	Modeling group	Validation group	*x* ^2^	*p*-value
		*n* = 140	*n* = 60		
Reason for admission	Spontaneous cerebral hemorrhage	61 (43.6%)	25 (41.7%)	0.377	0.539
	Traumatic cerebral hemorrhage	73 (51.14%)	34 (56.7%)	0.569	0.451
	Ischemic stroke	6 (4.3%)	1 (1.7%)	0.948	0.330
General Characteristics					
Age	>65 years old	54 (38.6%)	37 (61.2%)	0.005	0.944
	≤65 years old	87 (62.1%)	23 (38.8%)		
Gender	Male	114 (81.4%)	52 (86.7%)	0.407	0.524
	Female	26 (18.6%)	8 (13.3%)		
Smoking history	Yes	32 (22.9%)	19 (31.7%)	0.177	0.674
	No	109 (77.9%)	41 (68.3%)		
BMI	<18.5	22 (15.7%)	7 (11.7%)	0.123	0.94
	18.5 ~ 23.9	89 (63.6%)	35 (58.3%)		
	≥24.0	29 (20.7%)	18 (30.0%)		
APACHEIIScoring	<15	99 (70.7%)	38 (63.3%)	0.097	0.755
	≥15	41 (29.3%)	22 (36.7%)		
Surgical treatment	Yes	101 (72.1%)	47 (78.3%)	0.056	0.814
	No	39 (27.9%)	13 (21.7%)		
Past medical history					
Diabetes	Yes	33 (23.6%)	6 (10.0%)	0.581	0.446
	No	107 (76.4%)	54 (90.0%)		
Hypertension	Yes	92 (65.7%)	31 (51.7%)	0.013	0.911
	No	48 (34.3%)	29 (48.3%)		
Coronary heart disease	Yes	12 (8.6%)	7 (11.7%)	2.903	0.088
	No	128 (91.4%)	53 (88.3%)		
Cerebrovascular disease	Yes	13 (9.3%)	2 (3.3%)	0.082	0.774
	No	127 (90.7%)	58 (96.7%)		
Chronic pulmonary disease	Yes	15 (10.7%)	3 (5%)	0.004	0.949
	No	125 (90.3%)	57 (95.0%)		
ICU length of stay (days)	≤5	24 (17.1%)	10 (16.7%)	0.023	0.879
	6 ~ 10	53 (37.9%)	24 (40.0%)		
	11 ~ 15	34 (24.3%)	15 (25.0%)		
	≥15	29 (20.7%)	11 (18.3%)		
Total hospital stay (days)	≤10	14 (10.0%)	5 (8.3%)	0.304	0.581
	11 ~ 20	26 (18.6%)	10 (16.7%)		
	21 ~ 30	44 (31.4%)	19 (31.7%)		
	≥30	56 (40%)	26 (43.3%)		
Clinical outcome	Improved	77 (55.7%)	29 (48.3%)	3.329	0.066
	Unresolved	35 (25%)	18 (30.0%)		
	Deceased	28 (20%)	13 (21.7%)		

**Figure 2:**
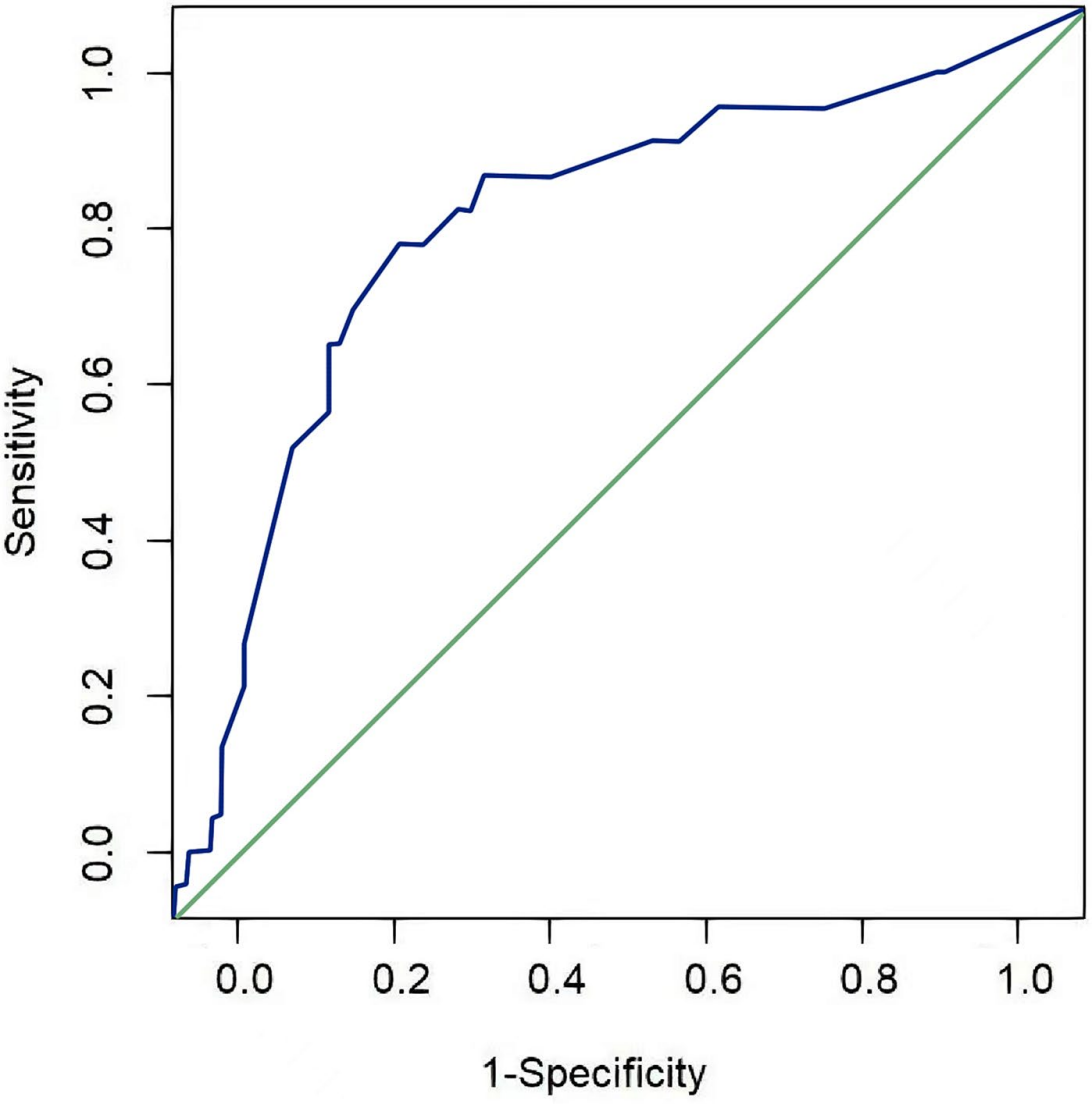
ROC curve of the risk prediction model in the validation group.

The variables of the validation group were input into the established risk prediction model of post-extubation dysphagia to calculate the risk of occurrence of dysphagia. The discriminative power and consistency of the prediction for the patients in the validation group were tested using the ROC curve method and the H-L goodness-of-fit test. The area under the curve (AUC) was 0.763 (95% CI, 0.652–0.875), as shown in Figure 2. The H-L goodness-of-fit test chi-square was 5.372, *p* = 0.717 (*p* > 0.05), suggesting that the established risk prediction model for extubation failure had good discriminative power and consistency. As shown in Table 9, the prediction results of the validation group indicate that the model has a high specificity of 84.8%, but a low sensitivity of 76.0%. The model’s negative predictive value is at a high level of 88.20%, while the positive predictive value is at a lower level of 59.20%. Overall, the model has a prediction accuracy of 79.8%. This indicates that the model can accurately predict the occurrence rate of extubation failure in ICU patients with severe neurological conditions who require tracheal intubation.

**Table 9:** Risk prediction model results validation.

Group	H-L test p-value	AUC	Sensitivity	Specificity	PPV	NPV	Predictive Accuracy
Modeling Group	0.732	0.832	84.10%	81.60%	66.30%	90.80%	81.50%
Validation Group	0.717	0.763	76.00%	84.80%	59.10%	88.20%	79.80%

AUC, Area Under the Curve, area under the curve; PPV, Positive Predictive Value, positive predictive value; NPV, Negative Predictive Value, negative predictive value.

## 4 Discussion

This study focuses on cases of patients with severe neurological diseases undergoing tracheal intubation. Through logistic multivariate regression analysis, eight independent risk factors were successfully screened out, including age, APACHE II score, chronic pulmonary disease, GCS score, oxygenation index, sputum volume, cough reflex, and swallowing function. A risk prediction model for extubation was constructed based on these factors and their partial regression coefficients. The study shows that the Logistic regression prediction model has a good fit, with both H-L goodness-of-fit test and ROC curve analysis indicating that the model has high discriminability and consistency, as well as high specificity, although the sensitivity is relatively low. The overall accuracy of prediction is high, with a high negative predictive rate and a low positive predictive rate, which may be related to the small number of positive samples. Therefore, future research needs to expand the sample size to further verify the performance of this prediction model. In addition, this study assigned scores to ICU tracheal intubation patients according to the scoring rules, and divided them into low-risk and high-risk groups, with 18 points as the critical value. The study found that the higher the score of the model, the higher the likelihood of successful extubation. This scoring model is easy to use, highly operable, and can effectively predict the risk of extubation failure in patients with traumatic brain injury and tracheal intubation, providing a strong basis for implementing individualized, targeted precision nursing, and preventing re-intubation and its complications.

The incidence of extubation failure in general critically ill patients is relatively high (10–15%), and in patients with neurological diseases, this proportion is even higher, reaching 25% (9, 11). In past studies on extubation in patients with severe neurological conditions, the extubation failure rate was approximately 21–25% (14, 21, 22). It is worth noting that there are differences in the definition of extubation failure across various studies, and to date, there is no consensus on the time frame for defining extubation failure. In recent years, some scholars have suggested extending the time range for extubation failure from 3 days after extubation to 7 days天 (23, 24). Miltiades et al. recommended using a time frame that captures more than 90% of extubation failures. Patients who underwent tracheotomy after attempting extubation failure were also included in this study (25).

We found that the rate of extubation failure was 25%, and the model score can predict successful extubation. Patients who underwent tracheotomy and those with extubation failure both showed longer invasive mechanical ventilation (IMV) times, higher rates of respiratory infections, and higher mortality rates. Vallverdu et al. (26) pointed out more than 20 years ago that safe delayed extubation is related to increased IMV time and hospital-acquired pneumonia for neurological recovery (26). In addition, we found that the independent risk factors affecting the success of extubation in neurocritical patients include age ≥ 65, APACHE II score ≥ 15, comorbid chronic lung disease, GCS score < 8, oxygenation index <300 on the day of extubation, frequent need for sputum suction due to excessive sputum, poor cough reflex, and comorbid dysphagia. It is worth noting that most patients in this study were intubated for neurological problems rather than respiratory diseases. Our study shows that the GCS score is an important indicator for assessing neurological conditions and is also an important factor in predicting successful extubation. Vallverdu and colleagues found in a prospective study that the re-intubation rate for patients with central nervous system (CNS) injuries was 36%, while Koh et al. observed a 22% re-intubation rate in a retrospective analysis. This difference may reflect higher standards for extubation and a higher frequency of tracheotomy (27). Our study supports the views of Epstein and Ciubotaru (1998), who identified several factors associated with increased risk of re-intubation, including non-airway-related causes, increased APACHE II score, and comorbid conditions. They also emphasized the importance of changes in neurological function and the characteristics of re-intubation due to extubation failure or weaning failure. Our study results also support the importance of this distinction. Although we took a conservative approach, only about 70% of the patients in our study were successful in the first attempt at extubation, in contrast to other populations in the ICU, where 85% of patients were successful in the first attempt at extubation (28, 29). Finally, although previous studies have reported that factors such as gender, BMI, and diabetes are related to the risk of extubation failure, these factors did not show statistical significance in our study (30–32). This may be related to our study subjects, screening criteria, and limited sample size. Although our study results are not entirely consistent with previous studies, they are still of great value for understanding and preventing extubation failure.

The novelties of this investigation are multifaceted: it is inceptioned from clinical conundrums with the aspiration to furnish innovative perspectives for their resolution. Currently, there is a paucity of systematic appraisals concerning the precision and comparative accuracy of extubation risk prognostication in patients with cranial injuries, precluding the ability to endorse any extant risk prediction models on the basis of empirical evidence. This study is underpinned by a theoretical ingenuity that is complemented by robust operational and practical attributes. Moreover, the research has led to the creation of an extubation risk prediction model and a scoring system through the quantification of risk factors associated with extubation failure, thereby undergoing rigorous validation, enhancing its operational and practical viability. It is anticipated that this model will aid in the prevention of various post-extubation complications in patients, thereby diminishing the rate of clinical extubation failures, and will hold significant clinical applicability.

Although our study has achieved positive results, there are some limitations to be noted. First, our sample size is small, which may affect the reliability and generalizability of the results. Second, our prediction model is based on data from a single center and may not be fully applicable to patient populations in other centers. Therefore, future research needs to be conducted with larger samples and at different centers to further establish the validity of our prediction model. The study’s cohort comprised merely six individuals with strokes, representing a notably limited sample size. Such a diminutive sample may precipitate less stable and dependable statistical analyses, given the vulnerability of small samples to chance factors. During the establishment and validation of the model, a petite sample size may fall short in encapsulating all variables influencing extubation outcomes, potentially compromising the model’s predictive capabilities. Owing to the restricted sample size and the singularity of the data source, the dependability of the findings may be compromised. Consequently, external validation across diverse patient cohorts is imperative. External validation serves primarily to substantiate the prevalence of various patient populations (33). Regional and institutional variations in patient demographics, disease severity, comorbidities, and treatment modalities may sway study outcomes. Future inquiries should endeavor to amass additional data, pursue multicenter investigations, and explore alternative methodologies to enhance the generalizability and dependability of the research findings. The marginally reduced sensitivity observed in the validation cohort warrants scrutiny and further exploration. In the context of clinical research, sensitivity is intrinsically linked to the efficacy of diagnostic tests or treatments. For diseases where early intervention is critical, decreased sensitivity may precipitate treatment delays, thereby influencing patient outcomes. With a fixed sample size, reduced sensitivity may diminish statistical power, thereby increasing the risk of type II errors (false negatives). In light of the study’**s** single-center design, it is crucial to replicate these findings across multicenter research settings to affirm the research conclusions. While our data delineated an association between extubation strategies and outcomes, the study’s framework precluded causal inferences. Data collection was confined to specific temporal points, such as the day of successful SBT or the day of tracheotomy. To facilitate data collection, we deliberately focused on pivotal clinical features pertinent to extubation day and ICU outcomes. Being an open-label study, we could not discount the potential influence of inter-clinician variability in extubation practices or adjust for patient management nuances. Furthermore, the study overlooked critical ICU-specific factors, including nurse-to-patient ratios, the availability of respiratory therapists, local protocols, and post-extubation management strategies (e.g., high-flow nasal catheter oxygen therapy). Notwithstanding, extubation procedures were conducted in adherence to clinical guidelines within this study. Lastly, the validation cohort was derived from the same sample as the training cohort, negating independence between the two. Therefore, our observations must be substantiated within an independent, prospective cohort.

Future scholarly endeavors ought to persevere in refining an efficacious and precise of extubation strategies, delineating unambiguous criteria for the commencement of extubation (upon the establishment of neurological stability and the resolution of respiratory insufficiency), delineating extubation protocols (encompassing spontaneous breathing trials of adequate duration), and specifying extubation criteria (entailing requirements for vomiting, swallowing efforts, coughing, and sputum aspiration) through formal evaluation. The utilization of non-invasive ventilation and high-flow oxygen in patients at elevated risk of extubation failure may constitute a beneficial approach for certain individuals with less compromised airway reflexes. In light of the ENIO findings and the outcomes of a recent randomized trial investigating early tracheotomy in ICU patients with severe cerebrovascular trauma, the avoidance of early direct tracheotomy may be warranted (15). In this context, although a higher level of consciousness has been associated with an increased likelihood of successful extubation in ENIO and other prior studies, it may not be imperative to extend invasive mechanical ventilation solely due to a persistent low Glasgow Coma Score if the patient’s neurological status is stable. Consequently, the safety of extubation in patients with diminished consciousness (inclusive of coma) warrants further investigation.

## Data availability statement

The original contributions presented in the study are included in the article/Supplementary material, further inquiries can be directed to the corresponding author.

## Ethics statement

The studies involving humans were approved by the Affiliated Hospital of Hangzhou Normal University. The studies were conducted in accordance with the local legislation and institutional requirements. The participants provided their written informed consent to participate in this study.

## Author contributions

WC: Writing – original draft. NZ: Writing – original draft. DL: Writing – original draft. HZ: Writing – original draft. LW: Writing – original draft. LL: Writing – original draft.
